# Social and material determinants of health in participants in an active labor market program in Barcelona

**DOI:** 10.1186/s13690-018-0310-4

**Published:** 2018-10-25

**Authors:** Patricia González-Marín, Vanessa Puig-Barrachina, Imma Cortès-Franch, Xavier Bartoll, Lucía Artazcoz, Davide Malmusi, Eva Clotet, Ferran Daban, Elia Díez, Àfrica Cardona, Carme Borrell

**Affiliations:** 1 Agència de Salut Pública de Barcelona, Plaça Lesseps, 1, Barcelona, Spain; 2 Centro de Investigación Biomédica en Red Epidemiología y Salud Pública (CIBERESP), Avenida Monforte de Lemos, 3-5 (Pabellón 11. Planta 0), Madrid, Spain; 3 Institut d’Investigació Biomèdica (IIB Sant Pau), Barcelona, Spain; 4 Departament de Pediatria, Obstetrícia i Ginecologia, i de Medicina Preventiva, Facultat de Medicina, Universitat Autònoma de Barcelona, Barcelona, Spain; 5 Universitat Pompeu Fabra, Barcelona, Spain; 6 Àrea de Drets Socials, Ajuntament de Barcelona, Carrer València, 344, 4a planta, Barcelona, Spain; 7 Barcelona Activa, Carrer de la Llacuna, 162-164 Barcelona, Spain

**Keywords:** Unemployment, Active labor market policy, Return to work program, Mental health, Self-rated health, Economic resources, Self-esteem, Social support

## Abstract

**Background:**

Unemployment affects the physical and mental health of affected individuals, which can be explained by its direct effect on worsening finances due to the lack of income as well as by its negative psychosocial effects. “Employment in the Neighborhoods” return to work program was implemented in Barcelona specifically in the neighborhoods characterized with a greater economic deprivation and by high unemployment to improve personal and occupational abilities and skills of the participants to reintegrate them into the workforce. The aim of this study is to determine the association between the lack of economic resources and psychosocial factors with respect to mental health and self-rated health in unemployed persons participating in the program “Employment in the Neighborhoods”.

**Methods:**

Cross-sectional study. Data collected from a self-administered questionnaire. Generalized linear models were constructed, adjusted by age and social class, to estimate prevalence ratios and analyze any possible association between economic resources, psychosocial factors and poor self-rated health and mental health.

**Results:**

Nine hundred forty-eight persons of 2763 participants in the “Employment in the Neighborhoods” program completed the questionnaire. 46.9% were women. 72.5% of women and 61.9% of men were at risk of poor mental health and 25.5% of women and 21.1% of men reported poor self-rated health. Low self-esteem [women: PR 1.88 95%CI (1.24–2.84); men: PR 2.51 95%CI (1.57–4.02)] and medium social support [2.01 (1.30–3.09)], in men, and low social support [1.74 (1.13–2.68)] in women are associated with worsening of self-rated health. In men, low self-esteem [1.40 (1.19–1.64)] and delay in paying bills [1.38 (1.17–1.64)] were associated with the risk of poor mental health; in women were associated low self-esteem [1.27 (1.11–1.44)] and received a non-contributory allowance [1.37 (1.09–1.74)].

**Conclusions:**

Economic resources, self-esteem and social support are necessary for good general and mental health among unemployed persons. The high prevalence of poor mental health among persons participating in the active labor market program “Employment in the Neighborhoods” could be due to a substantial deficit in these factors.

## Background

In the last decade, unemployment has markedly increased in some Europe countries, making it one of the main preoccupations in our society. Unemployment affects the physical and mental health of affected individuals [1–3], which can be explained by its direct effect on worsening individual and family finances due to the lack of income, as well as by its negative psychosocial effects.

The lack of economic resources affects the physical health of unemployed persons [1] and increases the risk of poor mental health [3]; however, the negative psychological effects of unemployment could be reduced by the ability to control economic difficulties [1]. Unemployment also negatively affects social relationships because employment, unless precarious, can promote social experiences within and beyond work [4]. Social support during unemployment is related to health [3, 5]. Huegarts [3] showed that unemployed women with low social support and men with medium support had a greater likelihood of poor mental health. Moreover, joblessness has also been related to a loss of self-esteem. Low self-esteem can generate stressful situations that are difficult to cope with partly due to the feeling of lack of control [6]. In addition, not having a job can generate a sense of failure, negatively affecting self-esteem; this can be aggravated if there is self-blame for the lack of employment. Unemployed persons with low self-esteem also feel that they have lost the social support of their friends during the period of unemployment but usually feel supported by their families [6]. Low social support can negatively affect the self-esteem of unemployed persons [5].

Prolonged unemployment negatively impacts the finances of affected individuals, worsening their physical and mental health [7]; in addition, it accentuates the relationship between social support and health [8] and can give rise to a permanent lack of self-esteem because, in addition to having feelings of failure and blame, unemployed people can feel excluded from society and believe that they will not be able to reintegrate into the workforce [9].

### Active labor market policies

Active labor market policies aim to improve the employability of unemployed persons. Studies show that these policies can, moreover, mitigate some of the negative psychosocial effects of unemployment. A study conducted in unemployed Finnish youth showed that taking part in an active labor market program helped them reestablish a daily routine and had increased their social support network. Moreover, participants reported they felt happier and more optimistic and had recovered belief in themselves and in the future [10]**.**

The “Employment in the Neighborhoods” project has been conducted in Barcelona since 2008. This project has been implemented in 13 neighborhoods of the city, specifically in the neighborhoods covered by the Neighborhood Law (Llei de Barris [Llei 2/2004]). These neighborhoods are characterized by greater economic deprivation than other neighborhoods in the city, as they have traditionally been assigned fewer resources, and by high unemployment. Part of the “Employment in the Neighborhoods” project consists of implementing a return to work program based on improving personal and occupational abilities and skills so that participants in the program can reintegrate into the workforce. Candidates for participation in the program have many difficulties in accessing the labor market.

The aim of this study was to determine the association between the lack of economic resources and psychosocial factors with respect to mental health and self-rated health in unemployed persons participating in the return to work program “Employment in the Neighborhoods”.

## Methods

### Design, information sources, study population

This is a cross-sectional study using data collected from self-administered ad hoc questionnaire developed to collect sociodemographic, health and quality of life of “Employment in the Neighborhoods” program participants.

The study population consisted of unemployed persons living in Barcelona who had participated in the “Employment in the Neighborhoods” program. A sample was chosen from persons participating between May 2015 and July 2016 and who had attended, at the very least, the information session of the program (445 women, 503 men).

Data collection was approved by the Clinical Research Ethics Committee of Parc de Salut Mar (Number 2015/6032 / I). Also, an informed consent to participate in the study was obtained from participants at the beginning of the study.

### Study variables

#### Outcomes

##### Self-rated health

This variable was obtained through the question “How is your health in general?” Possible responses were good (excellent, very good, and good) and poor (average or poor) [11].

##### Risk of poor mental health

This variable was measured with the 12-item General Health Questionnaire (GHQ12). Mental health was classified into good (GHQ12 < 3) and poor (GHQ12 ≥ 3). The scale was invalidated if responses were missing to 5 or more items [12].

#### Explanatory variables

##### Economic resources

These factors included the type of unemployment benefit, as well as delay in paying bills in the last year (housing, utilities, deferred payments). Unemployment benefit was categorized into: contributory allowance, non-contributory allowance (welfare benefit/subsidy and guaranteed minimum income) and no allowance. For delay in paying bills, a dichotomous variable (yes/no) was created, bearing in mind whether there had been a delay in paying for housing, utilities and/or purchases in the last year.

##### Psychosocial factors

These factors included social support and self-esteem. Social support was measured with the DUKE Social Support Index, which has been validated to Spain, and consists of 8 questions answered on a 5-point Likert format (1–5). The scale was invalidated if responses were missing to 3 or more items [13]. The variable was categorized into: low (1–2), medium (3) and high (4–5) social support. Self-esteem was measured by the Rosenberg scale, which has been validated to our environment, and is based on 10 questions answered on a 4-point Likert format (1–4) [14]. The variable was categorized into: normal (> 29) and low (<=29) self-esteem. No literature was identified on the number of missing values allowed in this scale; we considered the scale invalid if responses were missing to 4 or more items.

Other covariables were age, sex and social class. Age was measured continuously as a range from 16 to 64 years. Six categories were created for social class (I, II, III, IV, V, VI) based on the prior occupation of the interviewee or, if the person had never been in paid employment, on the highest-earning person in the household, following the neo-Weberian classification proposed by the Spanish Society of Epidemiology (CNO-11, Spanish acronym for National Classification of Occupations). This variable was classified into: non-manual (I, II, III), semi-manual and skilled manual (IV, V) and unskilled manual (VI) [15].

### Statistical analysis

First, the chi-square test was conducted to exclude a possible response bias among persons completing and those not completing the first questionnaire. Then, four generalized linear models were constructed, adjusted by age and social class, to estimate prevalence ratios and analyze any possible association between economic resources, psychosocial factors and poor self-rated health and mental health. Model 1 determined the association between explanatory variables and each of the outcomes. Model 2 examined the association between economic resources and poor self-rated and mental health. Model 3 identified the overall association between social support and economic resources with respect to the outcomes. Model 4 assessed the overall association between the explanatory variables and the outcomes. All analyses were stratified by sex.

Missing values were analyzed by multiple imputation by chained equations (MICE), following the rules of Rubin [16]. A total of 100 imputations from the database were conducted.

The STATA statistical program version 13 [17] was used.

## Results

Between May 2015 and July 2016, there were 2763 participants in the “Employment in the Neighborhoods” program. Of these, 948 completed the questionnaire and 46.9% were women. The mean age was 40.1 years (SD ± 0.51) in women and 41.2 years (SD ± 0.52) in men. Some statistically significant differences were observed between persons completing the questionnaire and those who did not; in our sample, there was over-representation of women not receiving unemployment benefit, men with secondary school education or less, and in both case persons who had been unemployed for less than 1 year.

Table 1 shows the participants’ sociodemographic, occupational and unemployment characteristics as well as their health status and quality of life. Most participants had secondary school education or less and were from a manual social class. A total of 45.6% of the women and 50.3% of the men had a temporary contract in their last employment. Forty-two percent of women and 51.9% of men had been unemployed for less than 1 year but 67.1% and 59.1%, respectively, received no unemployment benefit. Most participants (64.9% of women and 59.8% of men) reported some delay in paying their bills in the last year. In all, 72.5% of women and 61.9% of men were at risk of poor mental health and 25.5% of women and 21.1% of men reported poor self-rated health. Moreover, 44.2% of women and 46.4% of men had medium or low social support and almost 53% of women and men had low self-esteem.

**Table 1:** Participants’ sociodemographic, occupational and unemployment characteristics and health status and quality of life

	WOMEN (*N* = 445)	MEN (*N* = 503)
	Prevalence (%)	Prevalence (%)
Place of birth		
Outside the EU-15*	48.5	48.5
Spain and EU-15*	51.5	51.5
Education		
Primary school or less	48.3	55.9
Secondary school	41.3	36.2
University	10.4	7.9
Social class		
Unskilled manual	46.1	47.1
Skilled and semi-skilled manual	33.8	41.4
Non-manual	20.1	11.5
Type of work contract before unemployment		
Temporary	45.6	50.3
Permanent	27.7	26.6
Other	26.7	23.1
Type of unemployment benefit		
None	67.1	59.1
Non-contributory allowance	18.6	23.3
Contributory allowance	14.3	17.6
Length of time unemployed		
1 year or more	42.0	51.9
Less than 1 year	58.0	48.1
Payment of bills in the last year		
With delay	64.9	59.8
Without delay	35.1	40.2
Mental health		
Poor	72.5	61.9
Good	27.5	38.1
Self-rated health		
Poor	25.5	21.1
Good	74.5	78.9
Social support		
Low	18.3	19.8
Medium	25.9	26.6
High	55.8	53.6
Self-esteem		
Low	52.8	52.5
Normal	47.2	47.5

* plus Norway and Switzerland

Table 2 shows estimates of prevalence ratios of poor self-rated health by unemployment benefit, delay in paying bills, self-esteem and social support, adjusted by age and social class. Factors related to a worsening of self-rated health were delay in paying bills [women: PR 1.52 95%CI (1.02–2.27); men: PR 1.58 95% CI (1.05–2.40)], low self-esteem [women: 2.27 (1.53–3.37); men: 2.98 (1.87–4.75)] and medium social support [2.24 (1.46–3.42)], in men, and low social support [2.31 (1.54–3.45)] in women. In model 2, when delay in paying bills and type of unemployment benefit overall were introduced delay in paying bills lost statistical significance in women. When social support was introduced in model 3, the association was maintained between poor self-rated health and low social support [2.19 (1.46–3.29)] in women and medium social support [2.19 (1.43–3.36)] in men. When self-esteem was introduced in model 4, statistical significance was lost in the association between delay in paying bills and poor self-rated health in men.

**Table 2:** Association between economic and psychosocial variables and poor self-rated health

	WOMEN					MEN				
	Prevalence (%)	Models 1 PR (CI)	Model 2 PR (CI)	Model 3 PR (CI)	Model 4 PR (CI)	Prevalence (%)	Models 1 PR (CI)	Model 2 PR (CI)	Model 3 PR (CI)	Model 4 PR (CI)
Unemployment allowance										
Contributory allowance	20.8	1	1	1	1	19.5	1	1	1	1
Non-contributory allowance	33.1	1.63 (0.89–3.00)	1.51 (0.82–2.76)	1.45 (0.80–2.62)	1.79 (0.91–3.50)	24.3	1.22 (0.68–2.18)	1.17 (0.66–2.08)	1.10 (0.63–1.92)	1.10 (0.62–1.95)
None	24.4	1.29 (0.74–2.23)	1.25 (0.72–2.16)	1.25 (0.73–2.13)	1.49 (0.80–2.78)	20.3	1.15 (0.68–1.95)	1.10 (0.65–1.87)	1.05 (0.63–1.76)	1.08 (0.63–1.83)
Delay in paying bills in the last year										
No	19.0	1	1	1	1	15.5	1	1	1	1
Yes	29.0	1.52 (1.02–2.27)*	1.47 (0.98–2.20)	1.40 (0.94–2.08)	1.46 (0.97–2.20)	24.9	1.58 (1.05–2.40)*	1.57 (1.04–2.39)*	1.55 (1.03–2.35)*	1.46 (0.95–2.25)
Self-esteem										
Normal	15.0	1			1	10.2	1			1
Low	34.6	2.27 (1.53–3.37)***			1.88 (1.24–2.84)**	30.3	2.98 (1.87–4.75)***			2.51 (1.57–4.02)***
Social support										
High	17.8	1		1	1	13.8	1		1	1
Medium	29.8	1.64 (1.08–2.47)*		1.63 (1.09–2.45)*	1.43 (0.94–2.18)	31.7	2.24 (1.46–3.42)***		2.19 (1.43–3.36)***	2.01 (1.30–3.09)**
Low	43.1	2.31 (1.54–3.45)***		2.19 (1.46–3.29)***	1.74 (1.13–2.68)*	26.7	1.85 (1.14–3.00)*		1.86 (1.15–3.02)**	1.49 (0.88–2.51)

All models were adjusted by age and social class

*p-value < 0.05, **p-value < 0.01, ***p-value < 0.001

Models 1: Different models with the bivariate association between each explanatory variable and poor self-rated health

Model 2: Multivariate association between economic resources variables and poor self-rated health

Model 3: Multivariate association between economic resources variables, social support and poor self-rated health

Model 4: Multivariate association between all explanatory variables and poor self-rated health

Table 3 shows the estimates of the prevalence ratios of poor mental health by unemployment benefit, delay in paying bills, self-esteem and social support, adjusted by age and social class. The factors associated with the risk of poor mental health were delay in paying bills [women: 1.17 (1.02–1.35); men: 1.45 (1.22–1.72)], self-esteem [women: 1.35 (1.19–1.53); men: 1.60 (1.36–1.89)], medium social support [women: 1.34 (1.18–1.52); men: 1.60 (1.36–1.89)] and low social support [women: 1.25 (1.08–1.46); men: 1.59 (1.33–1.89)]; in women an association was found with receiving a non-contributory allowance [1.40 (1.11–1.78)]. When delay in paying bills and the type of unemployment benefit were introduced into the same model (model 2), the association between delay in paying bills and poor mental health was maintained in men [1.44 (1.21–1.71)] and was maintained in women if they received a non-contributory allowance [1.36 (1.08–1.73)]. Introducing social support to model 3 did not modify the association between economic variables (delay in paying bills and receiving benefits) and poor mental health in either sex. When self-esteem was introduced in model 4, the association between low social support and poor mental health lost statistical significance in women.

**Table 3:** Association between economic and psychosocial variables and poor mental health

	WOMEN					MEN				
	Prevalence (%)	Models 1 PR (CI)	Model 2 PR (CI)	Model 3 PR (CI)	Model 4 PR (CI)	Prevalence (%)	Models 1 PR (CI)	Model 2 PR (CI)	Model 3 PR (CI)	Model 4 PR (CI)
Unemployment benefit										
Contributory allowance	61.0	1	1	1	1	58.2	1	1	1	1
Non-contributory allowance	83.1	1.40 (1.11–1.78)**	1.36 (1.08–1.73)*	1.35 (1.07–1.70)*	1.37 (1.09–1.74)**	66.8	1.13 (0.90–1.43)	1.10 (0.87–1.38)	1.06 (0.85–1.32)	1.09 (0.87–1.36)
None	72.0	1.21 (0.97–1.52)	1.20 (0.96–1.51)	1.19 (0.96–1.48)	1.21 (0.97–1.51)	61.0	1.11 (0.89–1.38)	1.08 (0.87–1.33)	1.04 (0.85–1.27)	1.04 (0.85–1.28)
Delay in paying bills in the last year										
No	65.1	1	1	1	1	48.6	1	1	1	1
Yes	76.6	1.17 (1.02–1.35)*	1.14 (0.99–1.32)	1.14 (0.99–1.31)	1.12 (0.98–1.28)	71.0	1.45 (1.22–1.72)***	1.44 (1.21–1.71)***	1.43 (1.21–1.69)***	1.38 (1.17–1.64)***
Self-esteem										
Normal	62.1	1			1	46.6	1			1
Low	83.9	1.35 (1.19–1.53)***			1.27 (1.11–1.44)***	74.7	1.60 (1.36–1.89)***			1.40 (1.19–1.64)***
Social support										
High	64.1	1		1	1	48.0	1		1	1
Medium	85.4	1.34 (1.18–1.52)***		1.34 (1.18–1.52)***	1.25 (1.10–1.42)***	78.0	1.60 (1.36–1.89)***		1.58 (1.34–1.86)***	1.50 (1.27–1.78)***
Low	80.0	1.25 (1.08–1.46)**		1.21 (1.04–1.41)*	1.12 (0.97–1.31)	77.8	1.59 (1.33–1.89)***		1.59 (1.33–1.90)***	1.50 (1.25–1.79)***

All models were adjusted by age and social class

*p-value < 0.05, **p-value < 0.01, ***p-value < 0.001

Models 1: Different models with the bivariate association between each explanatory variable and poor mental health

Model 2: Multivariate association between economic resources variables and poor mental health

Model 3: Multivariate association between economic resources variables, social support and poor mental health

Model 4: Multivariate association between all explanatory variables and poor mental health

## Discussion

Persons participating in “Employment in the Neighborhoods” had substantial difficulties in returning to the labor market, which could negatively affect their health [1–3]. Participants had a high prevalence of poor mental health (72.5% of women and 61.9% of men) compared with the general population of unemployed persons in Barcelona (34.9% and 27.9%, respectively) [18]. Nevertheless, the prevalence of poor self-rated health (25.5% in women and 21.1% in men) was similar to that in the unemployed population in Barcelona (27.5% and 16.8%, respectively) [18]. These results agree with those of other studies conducted in disadvantaged populations, such as persons at risk of eviction, who also show a high prevalence of poor mental health compared with the general population [19, 20]. These studies show that extremes in lack of housing or work have a strong negative impact in this health indicator.

Difficulty in finding work led to prolonged unemployment in this collective; almost half of the participants had been unemployed for 1 year or more. Moreover, a high percentage of contracts were temporary. Both prolonged unemployment and temporary employment negatively affect the individual and/or family finances of these persons, reducing their ability to meet their basic expenses, which could give rise to debt accumulation; this, in turn, could increase the likelihood of poor mental health [19, 20] and poor self-rated health [7], mainly among men. Among women, an association was found between receiving a non-contributory allowance and worsening of mental and self-rated health. In Spain, non-contributory allowance are usually linked to temporary work and family responsibilities. It has been seen that in Europe, temporary contracts and informal workers are usually concentrated in women because they are the people who usually combine working and family life and caring for others. In Spain, care policies are deficient, and Spanish women usually have to play a greater role in providing informal care than in other countries with more effective policies. Because of the need to juggle these different roles, women have fewer work opportunities or their jobs are more precarious, which could increase poverty among women. From a gender perspective, the health impact of job precariousness is much higher in women than in men [21]. All this suggests that the occupational situation of women participating in the “Employment in the Neighborhoods” program is more precarious than that of men and that, moreover, they are the ones caring for children or elderly relatives.

Moreover, social support decreases when a person is unemployed [4], negatively affecting mental health [3, 5] and self-rated health in participants of the program. Unemployment also lowers self-esteem, which worsens health [6, 9]. Among participants in “Employment in the Neighborhoods”, self-esteem and social support were interrelated; having low self-esteem reduces a person’s social support [9] and, in turn, low social support negatively affects self-esteem [5]. This indicates that negative psychosocial factors in unemployed people, and specifically in the study sample, are closely linked to health; consequently, some of the high prevalence of poor mental health in this population could be explained by the lack of social support and low self-esteem.

Financial difficulties and, mainly, negative psychosocial factors such as low self-esteem and social support played a major role in worsening mental and self-rated health among participants in the “Employment in the Neighborhoods” program. These effects could be mitigated by improvements in employment policies, which could help unemployed persons cope with their situation more positively. This would, in turn, help them improve their health and reintegrate into the labor market.

### Limitations and strengths

The main limitation of this study is the response rate (36.2%). Nevertheless, the sample was sufficiently large for the study objectives and, moreover, there were no major differences between persons who completed the questionnaire and those who did not. Another limitation is inverse causality. This problem was avoided by excluding persons who terminated their last labor contract for health reasons. A final limitation is that self-esteem and social support are closely linked to mental health, which could have led to overestimation of some of the observed effects.

It is also necessary to keep in mind that the specific socioeconomic context, as well as different social positions along lines of gender, social class, ethnicity, age, migration status lead to specific experiences of unemployment with a differential impact on health [22]. Moreover, the impact of unemployment on health also depends on the unemployment rate, as well as the welfare state regimes and the state-level employment policies and regulations [23]. According to this, and due to the specificity of our population sample, our results are representative of “Employment in the Neighbourhoods” participants and cannot be generalized to other unemployed groups. However, these results were similar to those found in other contexts [3, 5, 6].

The main strength of this study is that it gathered information from a large number of persons participating in an active labor market program. This allowed us to identify the previous occupational situation of this collective and their current financial situation, as well as their health status and quality of life. Few studies with these characteristics have been conducted in Spain. Consequently, this study sheds light on a vulnerable collective and could prompt the performance of future, more specific and complex studies that could help to guide public policies to improve the health and quality of life of this population.

## Conclusions

Economic resources, self-esteem and social support are necessary for good general and mental health among unemployed persons. The high prevalence of poor mental health among persons participating in the active labor market program “Employment in the Neighborhoods”, compared with the unemployed population in Barcelona, could be due to a substantial deficit in these factors. The health of unemployed persons could be markedly enhanced by improving passive employment policies to alleviate the financial pressure on unemployed persons together with strengthening self-esteem and generating a new social network, through participation in active labor market programs such as “Employment in the Neighborhoods”.

## Abbreviations

CI: confidence interval
PR: prevalence ratio
SD: standard deviation
